# A revision of *Admetovis* Grote, with the description of a new species from western North America (Noctuidae, Noctuinae, Hadenini)

**DOI:** 10.3897/zookeys.788.26480

**Published:** 2018-10-08

**Authors:** Lars G. Crabo, B. Christian Schmidt

**Affiliations:** 1 724 14th Street, Bellingham, Washington 98225, USA; 2 Adjunct faculty: Washington State University, Pullman, Washington, USA; 3 Canadian National Collection of Insects, Arachnids, and Nematodes, Ottawa Research and Development Centre, Agriculture and Agri-Food Canada, Ottawa, Ontario, Canada K1A 0C6

**Keywords:** DNA barcode, Hadenini, Orthosiini, Taxonomy

## Abstract

The genus *Admetovis* Grote is revised. *Admetovisicarus***sp. n.** is described from the mountains of western North America. A lectotype of *Admetovisoxymorus* Grote is designated. Illustrations of the adults, male and female genitalia, and distribution maps are presented, together with an identification key. The classification of the genus is reviewed resulting in its reassignment to the tribe Hadenini from Orthosiini.

## Introduction

*Admetovis* Grote is a small genus of three western North American noctuid moths. The species are similar superficially, with a distinctive flame-like mark on the distal third of the otherwise gray forewing. Grote named the genus and first species in 1873. Two species, *Admetovisoxymorus* Grote and *Admetovissimilaris* Dyar, have been known since the turn of the last century. A third species from the Rocky Mountain region and Pacific Northwest resembling *Admetovisoxymorus* Grote was recognized recently. Its description is the main purpose of this article.

The higher classification of *Admetovis* is reviewed. Although the evidence is less than definitive, we recommend reclassification of this genus to the tribe Hadenini.

## Methods and materials

Wing pattern and genitalia structure terminology follow Lafontaine (2004). Forewing lengths are measured to the nearest half-millimeter from base to apex, excluding the fringe.

Male and female genitalia were prepared using standard methods (Hardwick 1950, Lafontaine 2004). Detached abdomens were macerated in hot 10 % KOH for 20–40 minutes. Dissection was performed initially in water or an ethanol-water mixture followed by hardening in isopropyl alcohol. The male vesicae and female bursae were inflated. Preparations were stained with orcein [Sigma Chemical Company, St. Louis, Missouri] and mounted in Euparal [Bioquip Products, Rancho Dominguez, California] on glass slides.

The 658 base pair DNA “barcode region” of the mitochondrial cytochrome *c* oxidase subunit 1 (CO1) (“DNA barcode”) was used to assess molecular variation. Legs from dried specimens were submitted to the Barcodes of Life Campaign (BOLD) at the University of Guelph (Ontario, Canada) where they were analyzed by standard DNA extraction, amplification, and sequencing protocols (Hebert et al. 2003). Barcode sequences were compared to pre-existing material at BOLD using the Kimura-2-Parameter distance model as implemented on the Barcode of Life Data System website (http://www.barcodinglife.org). The seven-unit BOLD Barcode Index Number (BIN) (Ratnasingham and Hebert 2013) is given in parentheses.

Distribution maps were made using SimpleMappr (http://simplemappr.net).

Repository abbreviations:

**AMNH** American Museum of Natural History, New York, New York, USA

**NHML** Natural History Museum, London, England (statutorily, British Museum of Nature History)

**CNC** Canadian National Collection of Insects, Arachnids, and Nematodes, Ottawa, Ontario, Canada

**JS** Jon Shepard Collection, Corvallis, Oregon, USA

**LGC** Lars Crabo Collection, Bellingham, Washington, USA

**OSAC** Oregon State Arthropod Collection, Oregon State University, Corvallis, Oregon, USA

**TM** Tomas Mustelin Collection, Seattle, Washington, USA

**USNM** Smithsonian Institution, Washington, D. C., USA

## Systematics

### Admetovis

Taxon classificationAnimaliaLepidopteraNoctuidae

Grote, 1873

#### Type species.

*Admetovisoxymorus* Grote, 1873 by monotypy.

#### Diagnosis.

**Adults.** Males and females similar in size and habitus, medium-sized (forewing length 17–21 mm) long-winged, stout noctuid moths; distinguished by combination of densely hairy eyes and gray forewing with a whitish, light tan, and reddish brown flame-like mark in both shape and colorfrom the medial reniform stigma to the jagged subterminal line. *Head* – Male antenna beaded weakly with slight constriction between segments, anterior, posterior, and ventral sides setose with innumerable short fine cilia; female antenna simple with few short lateral cilia. Scape whitish to gray tan, with ventral acute tuft. Eye normal size, densely setose. Labial palpus porrect, third segment length ~ ⅓ × second segment. Haustellum normal. Frons rounded, scales short, hair-like, forked, tan, brown, or gray brown. Dorsal head scales longer, forked, tan, gray, orange tan, sculpted weakly with paramedian dorsal and ventral protuberances. *Thorax* – Scales long, hair-like, forked, and scattered trifurcate, light tan, orange tan, and chestnut brown; appearing golden tan, with sculpted broad pale median tufts on mesothorax and metathorax and red-brown paramedian tufts on metathorax; prothoracic collar similar; appearing golden tan with pale-edged red-brown posterior band; tegula scales mostly white-tipped gray, forked; appearing gray with tan to red-brown medial edge. *Legs*: Lateral foretibia with row of 4–6 long, stout, slightly recurved, claw-like setae, proximal and distal longest; mid- and hindlegs lacking modified setae. Tarsal segments except apical segment with three rows of ventral spiniform setae. *Wings*: Forewing: Length 2.3–2.5 × width; apex pointed bluntly, outer margin slightly to moderately scalloped between veins; dorsal scales flat, fine toothed, elongate to triangular, white, gray, tan, and chestnut brown; ground color gray, darkest at costa and in cell, lightest at base; basal area posterior to vein 1A+2A light tan with anterior chestnut border; medial area distal to reniform spot and postmedial area whitish tan to tan, palest medially, forming with subterminal line and preceding chestnut shade a “flame” mark on distal ⅓ of wing; basal and antemedial lines double, gray with slightly lighter filling, undulating, perpendicular to wing axis; medial line dark gray, faint, diffuse; postmedial line thin, gray, faint, usually incomplete, scalloped strongly with points extended as thin dark lines and spots on veins, strongly oblique to wing axis from costa to posterior reniform stigma, less strongly to posterior margin; subterminal line thin, white to tan, bordered medially by prominent chestnut-brown and dark brown to black shade, darkest in fold, next darkest opposite reniform stigma, toothed to outer margin at apex and on M1, M3, CuA1, and 1A+2A, concave basad in fold; terminal line thin, black; fringe gray, striped, base pale tan or brown, pale transverse checkering at veins; claviform stigma dark gray to black, incomplete, apex darkest, short and broad, reaching mid-medial area, filling light gray; orbicular stigma black or chestnut brown, relatively large, ovoid to weakly figure-eight-shaped, filled variably with light gray, darker gray, light tan, and brown; reniform stigma moderately large, kidney shaped, usually with absent posterior and distal outline but evident due to whitish filling that contributes to palest portion of “flame” mark. Hindwing: Margin between M1 and M3 straight or concave; ground color pure white or slightly mottled light tan to brownish gray; veins, discal spot, and terminal line light to dark gray; fringe white to light gray with darker stripes, paler than ground. *Abdomen.* Male with or without basal coremata on segment I and pockets on ventrolateral segment III; when present weak, comprised of few filaments attached directly to base with pockets shallow, or strong, paint brush-like, filaments arising from rod with bulblike base, sometimes with very small medial accessory brushes, with pockets deep. Dorsal tufts on segments I–III. Female sternite VII sclerotized on distal half, with posterior median cleft 0.33–0.58 × segment length, blunt posterolateral lobes on side of cleft cover ostium bursae and part of ventral segment VIII. *Male genitalia*: Uncus relatively short, curved ventrad, base cylindrical, distal half widened and flattened dorso-ventrally, slightly concave dorsally with flange-like raised edges, tapering to truncate apex with small ventral spine; dorsal edges and ventral apex with numerous short setae. Juxta broadly shield shaped, wider than tall, smooth (*A.similaris*) or with median thorn-like spine directed posterodorsad (2 species). Valve length ~ 5 × width, S-shaped with slight curve dorsad near mid-point and 90° lateral bend distal to clasper at ⅔ distance from base to apex, tapered evenly from broad base to end of clasper, narrowing abruptly at bend to thin neck of cucullus, ventral margin of mid valve thickened with short blunt lateral projection ventral to distal clasper; sacculus length 0.4–0.5 × valve, sclerotized strongly, extending to or slightly above dorsal valve, distal costal margin near dorsal attachment of valve humped dorsad or anvil shaped, covered densely with minute setae; clasper strongly sclerotized, a bowl-like concavity dorsal and distal to sacculus, distal margin a narrow flange-like rim extended to blunt triangular dorsal and broad convex ventral projections, dorsal projection longer than (two species) or similar (one species) to ventral projection; digitus absent; cucullus triangular, 0.6–1.0 × mid-valve width, with slightly rounded margin bearing simple corona of 15–25 claw-like setae. Phallus tubular, length 6–7 × width. Vesica length (including right and left limbs) 1.0–1.5 × phallus, bent slightly ventrad at base then divided into nearly equal limbs directed 90° right and left to form with phallus a “T;” right limb comprised of distal vesica, tapered to ductus ejaculatorius at apex, with subapical broad short ventral diverticulum; left limb variable between species, tapering to conical or thin apical cornutus, bearing an additional thin acute cornutus at dorsal base (absent or diminutive in some specimens of all species) and anteriorly-directed broad conical subapical diverticulum (lacking in *A.icarus*). *Female genitalia*: Papilla analis asymmetrically conical, apex near dorsum, length 1–1.5 × width, covered sparsely on base and mid-portion by thin hair-like setae, more densely on apex by short setae. Abdominal segment VIII slightly longer than wide, venter length 2 × dorsum, covered sparsely by short hair-like setae; anterior apophyses length 0.57–0.77 × abdominal segment VIII; posterior apophyses length 2.75–3.35 × anterior apophyses. Ductus bursae length 1.14–1.32 × abdominal segment VIII, strongly sclerotized, flattened dorsoventrally, length ~ 3 × width, ventral surface smooth, dorsum rugose; ostium bursae simple. Corpus bursae length 3.9–4.6 × abdominal segment VIII, elongate, gourd shaped, anterior portion curved dorsad and leftward, membranous ovate anterior end widest, width 0.35–0.43 × bursa length, 3–4 longitudinal irregular string-of-beads signa evenly spaced on surface (left lateral signum absent in two species); appendix bursae projected ventrad and leftward from broad origin at junction with posterior corpus bursae at ductus bursae, length 0.12–0.25 × total corpus bursae length, sclerotized lightly, apex blunt, ductus seminalis at dorsum anterior to apex.

#### Distribution and ecology.

*Admetovis* species occur in western North America, from the Rocky Mountains and Arizona-Mexico border west to the Pacific Coast and north to southern British Columbia. They undoubtedly occur in Mexico but the distribution there is unknown.

The flight period of adults is from early spring (February or March) to as late as August depending on the species and locality. Based on limited information for *Admetovisoxymorus* the larvae feed on woody shrubs and are most likely climbing cutworms (McFarland 1975). All species in the genus are nocturnal and are attracted readily to light.

#### Discussion.

*Admetovis* was historically classified in the subfamily Hadeninae, where it was placed since the early twentieth century (Hampson 1905, McDunnough 1938, Hodges et al. 1982). It was reassigned recently to the tribe Noctuinae: Orthosiini (Lafontaine and Schmidt 2010) stemming from changes in the composition of the Noctuidae (Fibiger and Lafontaine 2005). Although the higher classification, including the recognition of Orthosiini as a tribe, is fairly recent (Fibiger and Lafontaine 2005) the relationship of *Admetovis* to other genera in this tribe is not obvious and had not been recognized widely. More recent molecular approaches to noctuid classification based on mtDNA barcode sequence data (Zahiri et al. 2017) support the current classification of North American Orthosiini in that nearly all included genera cluster together, but with the exception of *Admetovis* – this genus instead groups with genera in the Hadenini, which Godfrey also indicated as being the most closely related group to the orthosiine genera. This warrants a re-examination of Godfrey’s (1972) interpretation of larval hypopharynx structure, which forms the basis of the Orthosiini as currently defined.

Godfrey (1972) included nine genera in the *Orthosia* group (“Group 8”), noting that two other genus-groups were likely closely related based on the morphology of the hypopharynx, namely the spining pattern and especially the transverse cleft, the latter shared across the three groups. Godfrey’s three “transverse-cleft” groups comprise the *Anarta*-group, *Polia*-group and *Orthosia*-group, the first two now combined in the tribe Hadenini and the third comprising the Orthosiini. Although Godfrey separated the Orthosiini and Hadenini morphologically by two characters (lack of setae above the spinneret and larger spines on the proximolateral hypopharynx in Orthosiini), exceptions occur in both tribes (Godfrey 1972). *Admetovis* differs in the shape of the spinneret and the finely granular (vs. smooth) larval integument from all other Orthosiini, but these characters appear to be autapomorphic because they also do not occur in the Hadenini.

Importantly, *Admetovis* is the sole constituent of Godfrey’s Group 8 where adults do not emerge in early spring. Most species in the Orthosiini emerge very early in the season, and some, such as *Orthosiapraeses* (Grote) and *Egirahiemalis* (Grote), are the first non-overwintering moths to fly in late winter. While *A.similaris* can be found as early as February in the deserts of the Southwest, most *Admetovis* differ from other orthosiines in that they fly later in the year from late spring through summer.

Finally, the male valves of *Admetovis*, S-shaped with triangular cuculli, are more similar to those of most hadenine moths (e.g., as shown for European species in Hacker et al. 2002) than they are to orthosiines (shown similarly in Ronkay et al. 2001), as are the everted vesicae. Females of both tribes are rather simple in genital structure and as a result are not highly diagnostic.

The weight of evidence (barcodes, biology, and adult genitalia structure versus somewhat equivocal larva hypopharyngeal structure) suggests that *Admetovis* is better classified in the Hadenini rather than the Orthosiini. A definitive phylogeny of the Noctuinae tribes, using multiple molecular and morphological markers, is still needed. As such, we recommend *Admetovis* as a fertile subject for further investigation.

#### Key to species of *Admetovis* (adults)

**Table:** 

1	Dorsal hindwing pure white except thin gray veins and terminal line in some specimens; male juxta smooth, lacking central spine; female corpus bursae with four signa of nearly equal lengths	*** A. similaris ***
–	Hindwing off-white with tan or light gray mottling to gray brown, but not white; male juxta with spine-like median projection; female bursa with three signa, lacking one on left	**2**
2	Hindwing outer margin slightly concave between veins M1 and M3; dorsal hindwing light, tan off-white with scattered gray scales; male abdominal segment III with fully-developed coremata with rod-like base; female genitalia with relatively short appendix and bulbous anterior corpus bursae (corpus bursae width : appendix bursae length > 3.5)	*** A. oxymorus ***
–	Hindwing margin straight between veins M1 and M3; hindwing dark, brown gray; male abdominal segment III coremata vestigial, consisting of fine hairs (occasionally lost during genitalia preparation) without a rod; female genitalia with short appendix bursae and narrower corpus bursae (corpus bursae width : appendix bursae length < 2.5)	*** A. icarus ***

### Species accounts

#### Admetovis icarus sp. n.

Taxon classificationAnimaliaLepidopteraNoctuidae

http://zoobank.org/B293564D-4087-4F37-9BDE-FD3659627C15

**Figures 1–6.**
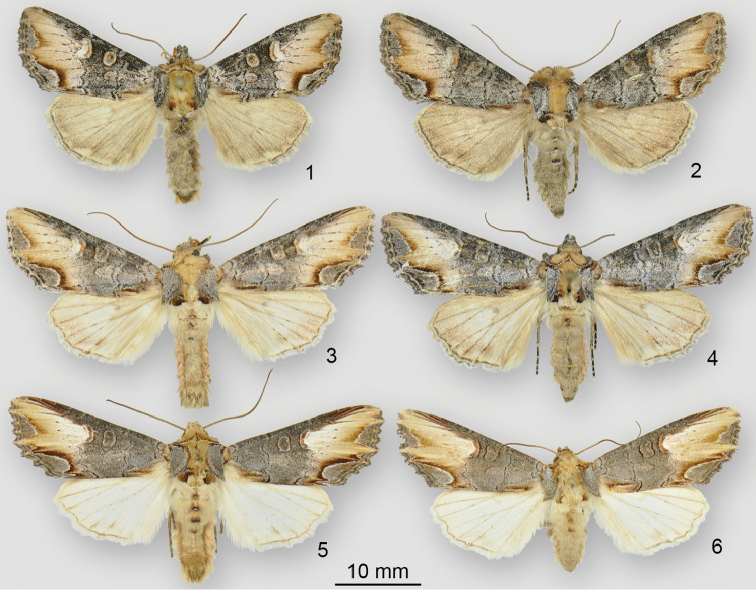
*Admetovis* adults. **1***A.icarus*, Holotype male, USA, Colorado, Boulder County, Nederland **2***A.icarus*, female, Canada, British Columbia, Apex Mountain **3***A.oxymorus*, male, USA, Oregon, Lane County, Frissell Point **4***A.oxymorus*, Neotype female, USA, California, Sierra County, Bassetts **5***A.similaris*, male, Washington, Kittitas County, Umtanum Creek/Durr Road **6***A.similaris*, female, USA, Washington, Walla Walla County, Walla Walla.

**Figures 7–12.**
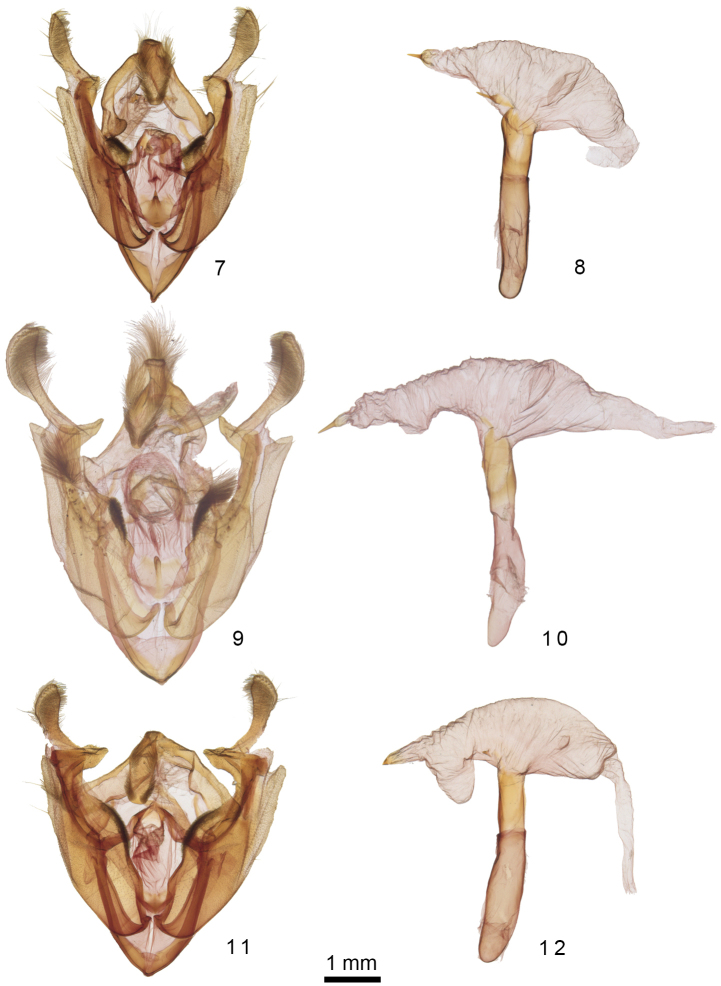
*Admetovis* male genitalia. **7***A.icarus*, valve*s***8***A.icarus*, phallus with everted vesica **9***A.oxymorus*, valves **10***A.oxymorus*, phallus with everted vesica **11***A.similaris*, valves **12***A.similaris*, phallus with everted vesica.

**Figures 13–15.**
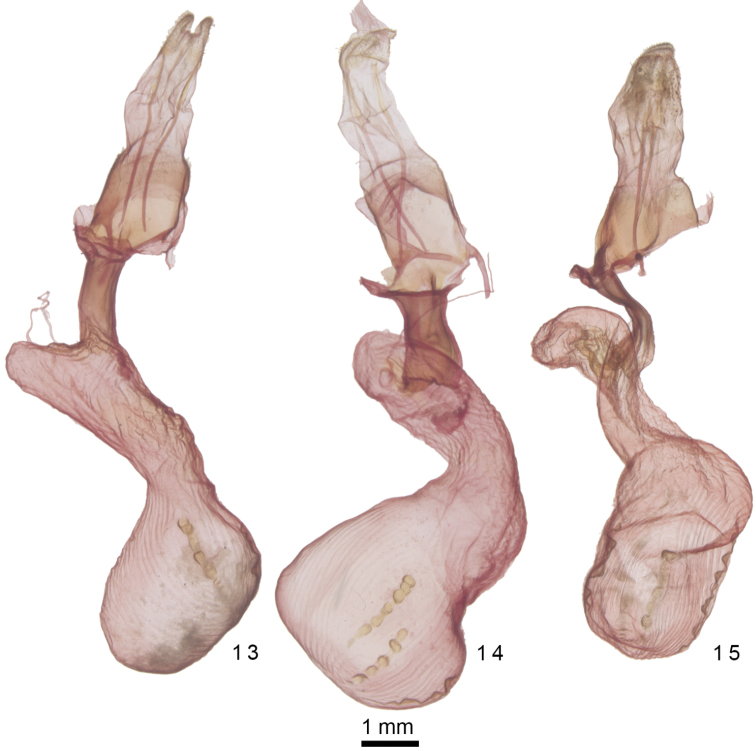
*Admetovis* female genitalia. **13***A.icarus***14***A.oxymorus***15***A.similaris*.

**Figures 16–17.**
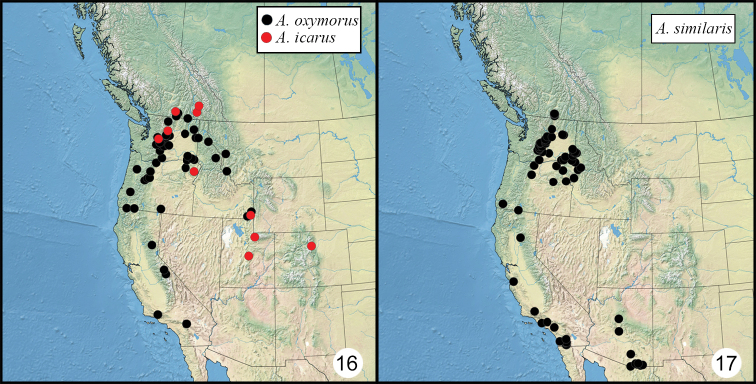
Distribution of examined material of *Admetovis* in western North America **16***A.icarus* (red) and *A.oxymorus* (black) **17***A.similaris*.

Figs 1 , 2 , 7 , 8 , 13 , 16

##### Type locality.

USA, Colorado, Boulder County, Nederland, 2896 m.

##### Type material.

**Holotype, male.** [USA]: Colorado: [Boulder County]: Nederland, Science Lodge, 9500’ [2896 m], 28 VI 1961, M. R. MacKay. CNC. **Paratypes.** 34 m 14 f: **CANADA: British Columbia**: Central Kootenay District: Sproule Cr., 49.533°, -117.417°, 2400’ [732 m], [no date], J. Shepard leg. / OSAC_0001031226 (1 m); [Okanagan-Similkameen District]: Apex Mt., 7380’[2249 m.], 49°21'N, 119°54'W, 21 VII 2000, J. Troubridge Leg. (1 f); **USA: Colorado**: [Boulder County]: Nederland, Science Lodge, 9500’ [2896 m], 26 VI 1961, M. R. MacKay (2 m); Same locality & collector, 27 VI 1961 (4 m 1 f); 28 VI 1961 (1 m 1 f); 29 VI 1961 (9 m 5 f); 30 VI 1961 (3 m 1 f); Same locality, 3 VII 1969, C. H. Mann (1 f); Gunnison County: Gothic, 29 VI 1962, Jon Shepard leg. / OSAC_0000136850 (1 m); Same locality, date, & collector / OSAC_0000136868 (1 f); **Idaho**: Bear Lake County: Emigration Cr. CG, 42.370°, -111.556°, 7200’ [2195 m], 14 VII 1993, J. & S. Shepard leg. / OSAC_0001031225 (1 m); **Oregon**: Wallowa County: Wallowa Mts., Mt. Howard summit area, 8075–8176 ft [2461–2492 m], 4 VIII 2016, UVBLT, DNR Ross and GE Pearson leg. / OSAC_0000997854 (1 m); **Utah**: Sanpete County: Ephraim, 8 mi. [12.9 km] E, 10,000’ [3048 m.], 39.317–[39].337°N, 111.448–[111].470°W, 21 VII 2008, L. G. Crabo leg. (8 m, 1 f); Same locality, date, & collector / DNA voucher # CNCLEP 00116343 (1 m); Summit County, Bald Mt. Trailhead, 14 VII 1989, R. C. Mower leg. / Database for CNCNoctuoidae [*sic*] 14629 [DNA voucher # NOC14629] / Barcodes of Life Project Leg removed DNA extracted (1 f); **Washington**: Chelan County: Junior Point Cmp. Grd., 6900’ [2103 m], 6 VIII 1997, J. Troubridge leg. (1 m); Same locality, date, & collector, DNA / Database for CNCNoctuoidae [*sic*] 14627 [DNA voucher # NOC14627] / Barcodes of Life Project Leg removed DNA extracted (1 m); Kittitas County: Lake Kachess (NF-4828), 47°19.21'N, 121°15.4'W, 4 VIII 2011, T. Mustelin (1 f). CNC, JS, LGC, OSAC, TM. Three specimens from Sandon, British Columbia (J. W. Cockle) at the CNC were also examined, but are excluded from the type series because they are worn.

##### Diagnosis.

*Admetovisicarus* is the only species in the genus with a dusky hindwing with a straight outer margin distal to the cell between veins M1 and M3. *Admetovisicarus* is distinguished easily from *A.similaris* by the pure white hindwing of the latter species. It is only subtly different from *A.oxymorus* in habitus and can easily be confused with it, especially in the Pacific Northwest where both species occur. While the hindwing margin shape is the most reliable character for separating these species short of dissection, there are subtle differences in color. *Admetovisicarus* tends be more mottled on the forewing with a darker “flame-mark,” and its hindwing is consistently darker brownish gray than that of *A.oxymorus*. The forewing orbicular stigma of *A.icarus* is often more conspicuous than in either other species, with pale filling outlining a dark central ocellus.

Structurally, males of *A.icarus* differ from both other species by the presence of weak basal abdominal coremata lacking a strong core; *A.oxymorus* has fully developed coremata with stout rods and *A.similaris* lacks them altogether. *Admetovisicarus* and *A.oxymorus* both differ from *A.similaris* in having a median spine on the juxta. In the valves, the setose dorsal protuberance of the sacculus is triangular to anvil shaped in *A.icarus*, convex in *A.oxymorus*, the dorsal process of the clasper is short and triangular in *A.icarus*, longer and curved in *A.oxymorus*, and the cucullus is relatively small and rounded in *A.icarus*, larger and triangular in *A.oxymorus*. The relative sizes of the cuculli are apparent readily if the two species are compared after the scales are removed with a brush. The vesica of the phallus of *A.icarus* is the simplest in the genus, with a relatively short left limb lacking a subapical diverticulum.

Females of *A.icarus*, like those of *A.oxymorus*, differ from those of *A.similaris* in having three rather than four signa on the corpus bursae. They can be differentiated from those of *A.oxymorus* by the shape of the corpus bursae, narrow with a small bulbous anterior portion in *A.icarus*; curved and broader with a bulbous anterior end in *A.oxymorus*. This difference is quantified in the Key as a ratio between width of the corpus bursae and the length of the appendix bursae.

The barcode of *A.icarus* (BOLD:AAD7456) differs from both other species by about 3.5 %. There is no intraspecific variation in three samples from Washington and Utah.

##### Description.

*Head* – Structure of male and female antennae, eye, palpus, and haustellum as for genus. Dorsal antenna tan with scattered gray scales. Scape off white. Labial palpus with nearly equal mixture of pale tan and dark gray scales. Frons tan, gray peripherally. Dorsal head scales white-tipped gray. *Thorax* – Dorsum as for genus; venter scales long, hair-like, white-tipped dark gray. Legs as for genus; tarsal segments dark gray banded distally with off-white. *Wings*: Forewing: length 16–17 mm (males), 17 mm (females), length 2.3–2.4 × width, outer margin scalloped weakly; ground lead gray, most mottled in genus due to more whitish scales, especially near base; medial area distal to reniform stigma and postmedial area whitish tan to tan, darkest distally; basal, antemedial, and medial lines as for genus; postmedial line dark gray, conspicuous for genus, double with strong inner and incomplete weak outer components, scalloped strongly with dark gray distal extensions on veins; postmedial white with preceding red-brown and black shade; terminal line black, interrupted at veins; fringe gray with light tan base, pale checkering at veins; claviform stigma dark gray, filling light gray; orbicular stigma nearly round, double, outer component darkest, filling light gray, whitish gray, or pale tan, central ocellus gray or mixed gray and brown; reniform stigma moderately large, kidney shaped with slightly larger posterior end, dark gray, incomplete at posterior end, filling of medial ⅓ light tan and distal ⅔ whitish tan or gray. Hindwing: margin straight between M1 and M3; powdery dusky gray brown with slight brassy sheen, discal spot and patchy marginal shade slightly darker with lighter patches near outer margin. *Abdomen.* Male coremata very weak, a few filaments arising from button-like base, lacking central stalk; pockets on ventrolateral segment III small, shallow. Female sternite VII posterior median notch 0.36 × length of the segment. *Male genitalia*: Uncus as for genus, wide distal portion only slightly tapered to blunt tip. Juxta height 1.5 × width, median spine present. Valve as for genus, length 5 × width, ventral mid-valve projection short, blunt; sacculus length ⅔ × valve, width 1 × valve, setose costal lobe asymmetric, anvil shaped with triangular pointed tip; clasper with short triangular dorsal and broad-based convex ventral processes of similar length, small for genus; cucullus relatively small, ~ 0.6 × valve width, rounded, corona 15–20 setae. Phallus tubular, length 6.8 × width. Vesica length 1.0 × phallus; diverticulum of right arm broad based, short; left arm tapered to straight spike-like apical cornutus, basal cornutus present or absent, diverticula absent. *Female genitalia*: papilla analis length 1.5 × width, longer and more pointed than in other *Admetovis*. Segment VIII as for genus; posterior apophysis length 0.75 × segment VIII; anterior apophysis length 2.75 × posterior apophyses. Ductus bursae relatively smooth, rugae limited to near junction with corpus bursae. Corpus bursae length 4.6 × abdominal segment VIII, elongate, narrow, curved weakly, bulbous anterior portion relatively small, diameter ~ 0.4 × total corpus bursae length; signa on dorsal, right lateral, and ventral sides; appendix bursae simple sac-like posterior extension of corpus bursae, length 0.16 × corpus bursae.

##### Geographic variation.

This species is fairly uniform throughout most of its range. The population on the Wasatch Plateau of central Utah is paler and more mottled than those from elsewhere.

##### Etymology.

The species epithet refers to Icarus, son of Daedalus in Greek mythology. Icarus used wings that his father had made to escape from the island of Crete but flew too close to the sun, thereby falling to his death in the sea. The flame marking on the distal forewing and high elevation habitat of this moth bring to mind his story. It is a noun in the genitive in apposition to the generic name.

##### Distribution and ecology.

*Admetovisicarus* occurs in the mountains of western North America, mostly in the Rocky Mountain region. Records extend from central Utah and central Colorado to the Selkirk Mountains of southeastern British Columbia, including a record from northeastern Oregon. Farther west there are scattered records from the Okanagan region of south-central British Columbia and Chelan and Kittitas counties in the northern Cascade Range, Washington. It replaces *A.oxymorus* in Utah and Colorado but is partially sympatric with it in the Pacific Northwest.

*Admetovisicarus* has been collected almost exclusively in high-elevation forests near tree line, although the habitat in Kaslo and Nelson, British Columbia is transition zone forest. Collection dates are from late June to early August.

The early stages are unknown.

##### Discussion.

*Admetovisicarus* is not rare in collections but has until now been confused with *A.oxymorus*.

#### Admetovis oxymorus

Taxon classificationAnimaliaLepidopteraNoctuidae

Grote, 1873

Figs 3 , 4 , 9 , 10 , 14 , 16

Admetovis oxymorus Grote, 1873: 133.

##### Type material.

*Admetovisoxymorus* was described from two syntypes, one each from Sierra Nevada and Rocky Mountains (Grote 1873), with an illustration of the California type on plate 4, figure 5. The depicted California female is clearly identifiable as the species that is widespread along the West Coast, an important distinction since based on current known distributions the other type specimen from the Rocky Mountains is most likely *A.icarus*.

We found neither type specimen in major collections known to contain Grote type specimens, including AMNH and NHML, and both specimens are most likely lost. This conclusion is supported by the fact that Poole (1989), in the Lepidopterorum Catalogus series, appears to not have examined the syntypes or known their whereabouts. He lists the type material noncommittally as “Type(s)” and cites “AMNH London [*sic*]” as the repository collection. In order to fix the identity of the name, we hereby designate a female specimen in the CNC labeled “CA, Sierra Co., 2 mi. E. Bassetts, 5300’ [1615 m], Hwy. 49, SNFC-SF State U., 4–7.Jul.2007, P. A. and E. Opler” as **Neotype** with a red label designating it as such.

##### Diagnosis.

This species and *A.icarus* have dorsal hindwings with gray or tan ground color and are distinguished easily from *A.similaris* that has a pure white hindwing with white or thin gray veins. *Admetovisoxymorus* is best distinguished from *A.icarus* by the shape of the hindwing. The margin between veins M1 and M3 is slightly concave in *A.oxymorus* but straight in *A.icarus*.

*Admetovisoxymorus* is the only species in the genus with fully developed male coremata; the coremata of the others are either vestigial or absent completely. The cucullus of the male valve of *A.oxymorus* is broadest in the genus, more than 2 × width of the adjacent neck versus less than 2 × in the other species. The relative sizes can be observed by brushing the scales from the end of the abdomen. Females of *A.oxymorus* have a bulbous anterior corpus bursae bearing three signa. Those of the other species either have four signa (*A.similaris*) or a smaller anterior bursa (*A.icarus*) as quantified in the Key to species.

The barcode of *A.oxymorus* (BOLD:AAD7455) differs from those of the other *Admetovis* species by slightly more than 3.5 %. Intraspecies variation within *A.oxymorus* is approximately 0.2 % (*n* = 7; British Columbia, California, Oregon).

##### Distribution and ecology.

*Admetovisoxymorus* occurs in the American West between the Rocky Mountains and Pacific Coast, as far north as extreme southern British Columbia. Most records are from the western part of this area, where it occurs throughout much of California, Oregon and Washington. The range is limited to Idaho and immediate vicinity in the Rocky Mountains.

*Admetovisoxymorus* is most commonly collected in hilly or mountainous areas with at least some trees, and occurs in a variety of habitats from riparian areas in steppe to near timberline.

Adults of *A.oxymorus* have been collected from late May until early August, with most records from mid-June through July. High-elevation populations fly latest, often in late July or August. It is nocturnal and comes to light.

*Admetovisoxymorus* is the only species in the genus for which the early stages are known. Reared larvae from southern California accepted elderberry (*Sambucusmexicanus* Presl.) in captivity (McFarland 1975). Godfrey (1972) illustrated the head and hypopharyngeal complex of the mature larva.

##### Discussion.

This species has until now been confused with *A.icarus*. Prior records of *A.oxymorus* from Utah and Colorado are referable to *A.icarus*.

#### Admetovis similaris

Taxon classificationAnimaliaLepidopteraNoctuidae

Barnes, 1904

Figs 5 , 6 , 11 , 13 , 15 , 17

Admetovis similis Barnes, in Dyar 1903: 157. *Nomen nudum*.
Admetovis similaris Barnes, 1904: 200.

##### Type material.

Three male and three female syntypes from Southern California and Arizona are at NMNH. All are typical of the species indicating that lectotype designation is unnecessary.

##### Diagnosis.

*Admetovissimilaris* is the easiest species in the genus to identify without examining structural characters. It is the only one in which the hindwing ground color is pure white.

Structurally, both sexes differ in several respects from those of the other two species. Males lack completely basal coremata, present to some degree in the other two. The juxta of *A.similaris* is smooth, lacking the median spine that is found in both other species. The left arm of the vesica bears the largest subapical diverticulum in the genus. Females have four signa on the corpus bursae, three in the other two species. The corpus bursae are similar otherwise to that of *A.oxymorus* in that the anterior end is bulbous, but the appendix bursae is more strongly curved in *A.similaris*.

The barcode of *A.similaris* (BOLD:AAB7673) differs from both other *Admetovis* species by at least 3.5 %. Intraspecies variation is less than 0.9 % (*n* = 19; Arizona, California, Washington).

##### Distribution and ecology.

*Admetovissimilaris* is a species of open habitats in the Southwest, California, and Pacific Northwest. It is found near the border with Mexico from western New Mexico to the coast of southern California, thence north to south-central British Columbia. Although its distribution is mostly in the region near the Pacific Coast it does not occur near the ocean north of the San Francisco Bay area. In the Pacific Northwest *A.similaris* is common on the Columbia Plateau, in the adjoining Cascade Foothills, and at low elevations in the Blue Mountains. Interestingly, it is absent from similar steppe habitats in southeastern Oregon and southern Idaho and it does not occur elsewhere in the Great Basin. *Admetovissimilaris* almost certainly occurs in Mexico as it is found very close to the Mexican border both in Arizona and in California.

This species favors the most xeric environments of any *Admetovis*, as dry as the Sonora and Mojave deserts. Northern populations fly most commonly in sage steppe. The flight time is during spring and early summer, typically earlier in the year than either of the other two species.

The early stages are unknown.

##### Geographic variation.

The color and pattern of this moth are uniform across its range. Specimens from deserts of the Southwest tend to smaller than those from elsewhere.

##### Discussion.

Dyar (1903) included this species in his list of North American Lepidoptera as *Admetovissimilis* prior to its proper description by Barnes in 1904. The Dyar mention lacks a description or illustration and is therefore a *nomen nudum*.

## Supplementary Material

XML Treatment for Admetovis

XML Treatment for Admetovis icarus

XML Treatment for Admetovis oxymorus

XML Treatment for Admetovis similaris
